# Photodehydrogenation of Ethanol over Cu_2_O/TiO_2_ Heterostructures

**DOI:** 10.3390/nano11061399

**Published:** 2021-05-25

**Authors:** Congcong Xing, Yu Zhang, Yongpeng Liu, Xiang Wang, Junshan Li, Paulina R. Martínez-Alanis, Maria Chiara Spadaro, Pablo Guardia, Jordi Arbiol, Jordi Llorca, Andreu Cabot

**Affiliations:** 1 Catalonia Institute for Energy Research (IREC), Sant Adrià de Besòs, 08930 Barcelona, Spain; congcongxing@irec.cat (C.X.); peterzhang@irec.cat (Y.Z.); wxiang@irec.cat (X.W.); junshanli@irec.cat (J.L.); pguardia@irec.cat (P.G.); 2 Institute of Energy Technologies, Department of Chemical Engineering and Barcelona Research Center in Multiscale Science and Engineering, Universitat Politècnica de Catalunya, EEBE, 08019 Barcelona, Spain; 3 Laboratory for Molecular Engineering of Optoelectronic Nanomaterials (LIMNO), École Polytechnique Fédérale de Lausanne (EPFL), Station 6, CH-1015 Lausanne, Switzerland; yongpeng.liu@epfl.ch; 4 ENFOCAT-IN2UB, Universitat de Barcelona (UB), C/Martí i Franquès 1, 08028 Barcelona, Spain; paulina.martinez@ub.edu; 5 Catalan Institute of Nanoscience and Nanotechnology (ICN2), CSIC and BIST, Campus UAB, Bellaterra, 08193 Barcelona, Spain; mariachiara.spadaro@icn2.cat (M.C.S.); jordi.arbiol@icn2.cat (J.A.); 6 ICREA, Pg. Lluis Companys 23, 08010 Barcelona, Spain

**Keywords:** titanium dioxide, copper oxide, photodehydrogenation, ethanol, thermo-photocatalysis, hydrogen

## Abstract

The photodehydrogenation of ethanol is a sustainable and potentially cost-effective strategy to produce hydrogen and acetaldehyde from renewable resources. The optimization of this process requires the use of highly active, stable and selective photocatalytic materials based on abundant elements and the proper adjustment of the reaction conditions, including temperature. In this work, Cu_2_O-TiO_2_ type-II heterojunctions with different Cu_2_O amounts are obtained by a one-pot hydrothermal method. The structural and chemical properties of the produced materials and their activity toward ethanol photodehydrogenation under UV and visible light illumination are evaluated. The Cu_2_O-TiO_2_ photocatalysts exhibit a high selectivity toward acetaldehyde production and up to tenfold higher hydrogen evolution rates compared to bare TiO_2_. We further discern here the influence of temperature and visible light absorption on the photocatalytic performance. Our results point toward the combination of energy sources in thermo-photocatalytic reactors as an efficient strategy for solar energy conversion.

## 1. Introduction

Molecular hydrogen, a clean energy carrier and a key component in the chemical industry, is mostly produced through partial oxidation and steam reforming of natural gas and coal gasification. To move away from the exploitation of fossil fuels, cost- and energy-effective strategies for the direct production of hydrogen from renewable sources need to be defined. In this context, biomass resources are a particularly compelling alternative source of hydrogen owing to their renewable character and their near net-zero CO_2_ footprint [1,2,3,4,5,6]. Additional advantages of the hydrogen production from dehydrogenation of biomass-derived organics are the potential to co-produce valuable side organic chemicals for better process economics and the possibility to implement cost-effective waste abatement processes [7,8].

Among the possible dehydrogenation processes, photocatalytic routes that make use of ubiquitous, abundant and renewable solar energy are especially attractive. Photocatalytic processes also enable the dehydrogenation reaction to take place in milder conditions, which further decreases costs and can increase the side product selectivity compared with thermocatalytic analogs [2]. From another point of view, the photocatalytic production of fuels can be considered as a convenient strategy to store intermittent solar energy [9,10].

In this scenario, the photodehydrogenation of ethanol to produce molecular hydrogen and acetaldehyde using solar light as the only energy input is especially appealing [6,11]. As a liquid, ethanol can be easily stored and transported. Besides, ethanol can be easily produced from several biomass-derived feedstocks and organic residues such as sewage sludge [12,13,14]. Additionally, bioethanol aqueous solutions can be directly used, without the need for purification. Compared with water splitting, the production of hydrogen from ethanol is thermodynamically advantageous (∆G^0^ = +237 kJ·mol^−1^ for water oxidation vs. ∆G^0^ = +41.5 kJ·mol^−1^ for ethanol oxidation to acetaldehyde), which decreases the energy input required to drive hydrogen production [2,8]. Compared with water splitting, ethanol dehydrogenation also enables a much simpler product purification, preventing the H_2_ and O_2_ back reaction. Besides, compared with ethanol photoreforming, ethanol photodehydrogenation to H_2_ and acetaldehyde could have a threefold higher economical profitability associated with the high economic value of the side product [15].

In terms of catalysts, while photocatalytic water splitting requires semiconductors with conduction and valence band edges sufficiently above and below the potentials for H^+^ reduction and water oxidation, respectively, ethanol dehydrogenation can be activated in semiconductors with significantly lower band gaps. On the other hand, the catalytic dehydrogenation of ethanol competes with the deoxygenation, reforming and decomposition reactions, which makes the selectivity of the catalytic process fundamental to ensure cost-effectiveness [1].

Copper oxides, Cu_2−x_O, have raised increasing attention as photocatalytic materials owing to their abundance, low cost, minor environmental and health impact and suitable optoelectronic properties. Cu_2−x_O are p-type semiconductors with a very energetic conduction band and a relatively low bandgap: 2.1 eV for Cu_2_O and 1.2 eV for CuO, which enables absorption of the visible range of the solar spectra. As a drawback, Cu_2−x_O have poor photostability, being prone to photocorrosion in reaction conditions. Besides, Cu_2−x_O generally presents a large defect density that results in a relatively fast recombination of photogenerated charge carriers. To solve these limitations, Cu_2−x_O can be combined with TiO_2_ within p-n heterojunctions that protect Cu_2−x_O against photocorrosion and reduce the charge carrier recombination. The synergism between the two materials is enabled by the appropriate conduction band edges of Cu_2−x_O, −1.79 V for Cu_2_O and −1.03 V for CuO, which allows the rapid injection of the photogenerated electrons from the Cu_2−x_O to the TiO_2_ conduction band [8,15,16,17,18,19]. Thus, the combination of Cu_2−x_O and TiO_2_ is regarded as a highly interesting photocatalyst to: (i) stabilize the Cu_2−x_O, (ii) boost the overall catalytic activity by extending the light absorption of TiO_2_ toward the visible light range and (iii) maximize external quantum yield by a rapid charge separation between the two phases enabled by their adequate band edges.

While the concept of a p-n heterojunction between Cu_2−x_O and TiO_2_ that promotes catalytic activity is pleasantly simple, real systems are much more complex, and Cu_2−x_O have been reported to promote catalytic activity through several different mechanisms: (i) Cu_2−x_O can absorb the visible light and transfer photogenerated electrons to TiO_2_, where H_2_ evolves, while using photogenerated holes to oxidize the organic species [20]. (ii) Cu_2−x_O can absorb visible light but use photogenerated electrons to evolve H_2_ and recombine in photogenerated holes at the Cu_2−x_O/TiO_2_ interphase within a Z-scheme mechanism [21]. (iii) Cu_2−x_O nanoparticles can be reduced to metallic copper during ethanol photodehydrogenation, and the resulting metal nanoparticles can act as a cocatalyst, stabilizing photogenerated electrons, promoting the water reduction reaction, simultaneously reducing the rate of charge recombination and, thus, also making more holes available for the oxidation reaction [15,22,23]. (iv) Cu^δ+^ and Cu^0^ on the surface of supported Cu clusters can also participate as catalysts in the ethanol oxidation to acetaldehyde [17]. (v) Copper ions can be partially incorporated into the TiO_2_ lattice by substituting for Ti^4+^ ions and creating oxygen vacancies that decrease the TiO_2_ bandgap [15,19,24,25]. All these effects strongly depend on the synthesis procedure, the TiO_2_ surface area and its structural and chemical properties, which affect the Cu dispersion and oxidation states [22] and the TiO_2_ phase that also determines the interaction with Cu and the Cu role [26].

Most previous works assign the performance promotion of Cu_2−x_O/TiO_2_ with respect to TiO_2_ to the extension of light absorption toward the visible range of the solar spectra. However, in most previous works, mainly UV excitation is used, and the overall and local temperature changes associated with the visible light absorption are usually neglected.

In the present work, we aim at gaining additional understanding of the mechanism behind the synergistic promotion of the catalytic performance in Cu_2_O/TiO_2_ while simultaneously contributing to the optimization of this system. In this direction, we present a one-pot hydrothermal synthesis strategy to produce Cu_2_O/TiO_2_ nanocomposites with controlled Cu_2_O amounts. The photocatalytic performance of Cu_2_O/TiO_2_ toward ethanol dehydrogenation is tested using both UV and visible light irradiation. We then determined the direct contribution of visible light, beyond the increasing temperature, toward increasing catalytic activity. We tested photocatalytic activity in the gas phase as it offers additional advantages, including lower light scattering, easier scale-up, higher stability, easier product recovery and even higher selectivity [7,15]. Besides, using time-resolved photoluminescence measurements and analyzing the band alignment between the two materials, we showed the activity promotion to proceed through a conventional p-n type II heterojunction.

## 2. Materials and Methods

### 2.1. Chemicals

Titanium (IV) isopropoxide (97%, Sigma-Aldrich, St. Louis, MO, USA), copper (II) nitrate hexahydrate (98%, Fluka, Buchs, Switzerland), ethanol (96%, PanReac AppliChem GmBH, Darmstadt, Germany), polyvinylpyrrolidone (PVP, 90%, Sigma-Aldrich, St. Louis, MO, USA), and sodium sulfate (Alfa Aesar™, Ward Hill, MA, USA) were used without further purification.

### 2.2. Synthesis of Photocatalysts

PVP (0.45 g) was dissolved in Milli-Q water:ethanol (1:2) (40 mL) under stirring at room temperature. To this solution, a proper amount of Cu (NO_3_)_2_·6H_2_O was added (0, 11.3, 22.5, 45 and 112.5 mg to reach 0%, 0.5%, 1%, 2% and 5%, respectively) by stirring for 5 min. Then, 2.3 mL of titanium (IV) isopropoxide was added dropwise, followed by stirring for 10 h at room temperature. Finally, the suspension was transferred to a 50-mL Teflon-lined autoclave and maintained at 170 °C for 14 h.

### 2.3. Structural and Chemical Characterization

The morphology and size of the particles were obtained by transmission electron microscopy (TEM) using a ZEISS LIBRA 120 (Carl Zeiss, Jena, Germany) instrument. Elemental analysis was carried out using an Oxford energy dispersive X-ray spectrometer (EDX) combined with the Zeiss Auriga SEM (Carl Zeiss, Jena, Germany) working at 20.0 kV. The crystal structure of the samples was determined by X-ray diffraction (XRD) using a D8 Advance (Bruker, Billerica, MA, USA) equipment with Ni-filtered Cu-Kα radiation (λ = 0.15406 Å) operating at 40 mA and 40 kV. UV-Vis absorption spectra were recorded on a UV-Vis spectrophotometer (Shimadzu, UV-3600i Plus, Tokyo, Japan), and BaSO_4_ was used as a reference standard. The spectra were recorded at room temperature in the air within the range of 300–800 nm. High-resolution transmission electron microscopy (HRTEM) images and scanning transmission electron microscopy (STEM) studies were conducted on an FEI Tecnai F20 field emission gun microscope operated at 200 kV with a point-to-point resolution of 0.19 nm, which was equipped with high angle annular dark-field (HAADF) and a Gatan Quantum electron energy loss spectroscopy (EELS) detectors. X-ray photoelectron spectroscopy (XPS) was done on a SPECS system (SPECS GmbH, Berlin, Germany) equipped with an Al anode XR50 source operating at 150 mW and a Phoibos 150 MCD-9 detector (SPECS GmbH, Berlin, Germany). Data processing was performed with the CasaXPS program (Casa Software Ltd., Teignmouth, UK). Steady-state photoluminescence (PL) spectra were conducted by a high-resolution photoluminescence spectrofluorometer (Horiba Jobin Yvon Fluorolog-3, Palaiseau, France). For the time-resolved photoluminescence spectroscopy (TRPL) measurements, a nanosecond LED with a 350-nm peak wavelength (Horiba NanoLED N390, Palaiseau, France, pulse width < 1.3 ns) was applied to excite the samples. The TRPL decay was resolved at 400 nm. Average lifetimes were obtained by fitting the TPPL spectra with DAS6 software (Horiba, Palaiseau, France).

### 2.4. Photoelectrochemical Measurements

Photoelectrochemical (PEC) properties were measured using CHI760e (CHI 760E, CH Instrument, Austin TX, USA) in a three-electrode cell with a platinum mesh as the counter electrode, and an Ag/AgCl reference electrode. Na_2_SO_4_ (0.5 M) was used as the electrolyte solution. The working electrode was prepared by depositing Cu_2_O/TiO_2_ on an indium tin oxide (ITO) glass electrode (1 cm × 1 cm) and heating at 200 °C for 1 h. Potentials vs. Ag/AgCl were converted into potentials vs. reversible hydrogen electrodes (RHE), according to the Nernst equation (E_RHE_ = E_Ag/AgCl_ + 0.059 pH + 0.196). Electrochemical impedance spectroscopy (EIS) measurements were carried out with a sinusoidal ac perturbation of 5 mV applied over the frequency range of 0.01–100,000 Hz. The transient photocurrent (TPC) of the as-prepared photocatalysts was measured with an AM1.5G solar power system used as the light irradiation source (100 mW·cm^−2^) at an ambient temperature and without any light irradiation source. Mott–Schottky (M–S) measurements were carried out in the dark with a scanning speed of bias potential ranging from −1.4 to 0.2 V at a scan rate of 0.01 V∙s^−1^. The linear sweep voltammetry was carried out with a scanning speed of bias potential ranging from −1.2 to 0.6 V at a scan rate of 0.01 V∙s^−1^.

### 2.5. Photocatalytic Test

In a typical experiment, a cellulose paper impregnated with 2.0 mg of the photocatalyst was placed inside a photocatalytic reactor that was equipped with UV LEDs (365 ± 5 nm, from SACOPA S.A.U, Gerona, Spain) (Figure S1). A light irradiation of 79.1 ± 0.5 mW·cm^−2^ was measured for UV light at the sample position. A saturated Ar gas stream was prepared by bubbling dry Ar gas through a Dreschel bottle with a water:ethanol vapor mixture (9:1, molar ratio, 20 mL∙min^−1^). The photoreactor effluent was monitored online every 4 min using gas chromatography (GC) (Agilent 3000A MicroGC, Santa Clara, CA, USA) with three columns: MS 5 Å, Plot U and Stabilwax. The system was purged with the saturated Ar stream (20 mL∙min^−1^, 30 min) to remove oxygen before performing the experiments. The UV-visible light source contained two LEDs emitting at 372 ± 5 nm and two LEDs emitting visible light (correlated color temperature (CCT) 6099 K and color rendering index (CRI) 74) in Figure S1. In this system, UV light irradiation was 11.2 ± 0.5 mW·cm^−2^ at the sample position.

### 2.6. Apparent Quantum Yield (AQY) Calculation

The AQY was estimated using the following equation:

(1) $$\mathrm{AQY}=\frac{2n_{H_{2}}}{\begin{array}{c} n_{p} \end{array}}\cdot 100=\frac{2{\mathrm{nN}}_{A}}{\begin{array}{c} E_{T}/E_{p} \end{array}}\cdot 100$$

where $n_{H_{2}}$ is the number of evolved hydrogen molecules, and n_p_ is the number of incident photons reaching the catalyst. The number of incident photons can be calculated by $n_{p}=E_{T}/E_{p}$, where E_T_ is the total energy reaching the catalyst, and E_p_ is the energy of a photon. E_T_ can be calculated by $E_{T}=\mathrm{PSt}$, where P (W·m^−2^) is the power density of the incident monochromatic light, S (m^2^) is the irradiation area and t (s) is the duration of the incident light exposure. E_p_ can be calculated by $E_{p}=\mathrm{hc}/\lambda$, where h is the Planck’s constant, c the speed of light and λ (m) is the wavelength of the incident monochromatic light. The number of hydrogen molecules can be calculated as $n_{H_{2}}={\mathrm{nN}}_{A}$, where n is the H_2_ moles evolved during the time of light exposure (t), and N_A_ is the Avogadro constant. In our experimental conditions with UV light, the wavelength of the incident light was λ = 365 nm, the power density of the incident light at the paper surface was P = 79.1 mW·cm^−2^ and the irradiation area was S = πR^2^ = 3.14 × 0.75^2^ = 1.77 cm^2^.

## 3. Results and Discussion

### 3.1. Structural, Chemical and Optical Properties

**Figure 1:**
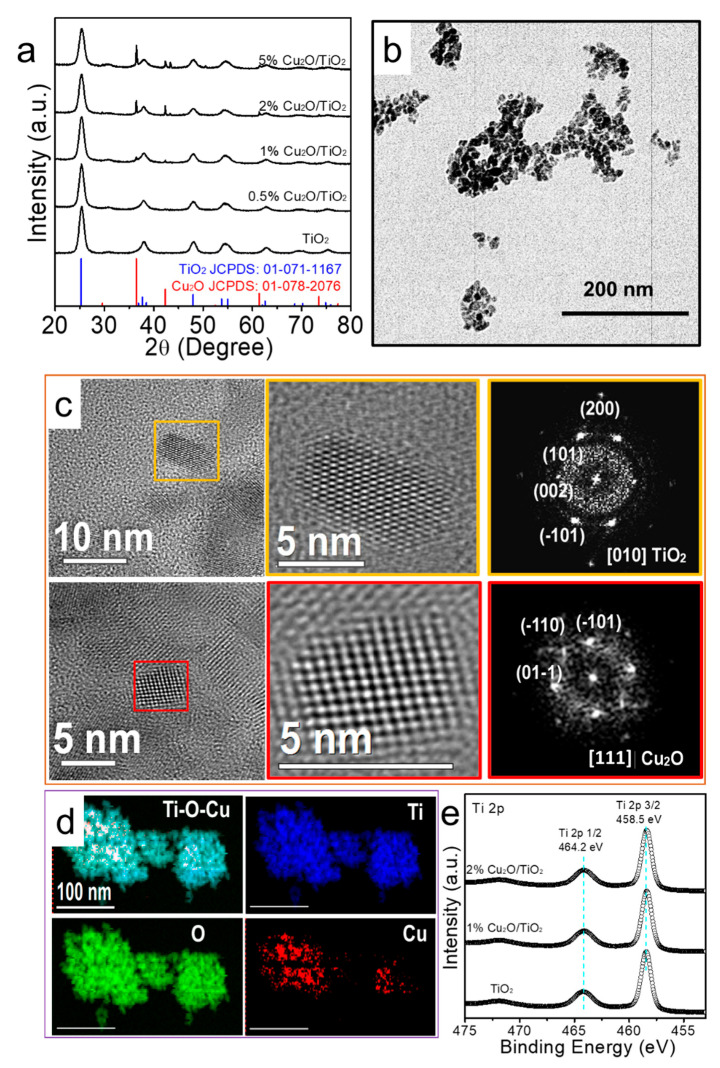
(**a**) Powder XRD pattern of TiO2 and 0.5%, 1%, 2% and 5% Cu2O/TiO2 nanocomposites. (**b**) TEM micrograph of 1% Cu2O/TiO2, with a scale bar of 200nm. (**c**) HRTEM analysis of the 1% Cu2O/TiO2 sample. The upper image shows a crystal with a tetragonal anatase phase of TiO2 visualized along the [010] zone axis. The lower image shows a cubic Cu2O crystallite visualized along the [111] zone axis. (**d**) STEM-ADF and STEM-EELS analysis of the 1% Cu2O/TiO2 sample. Cu L-edges at 931 eV (red), O K-edge at 532 eV (green) and Ti L-edge at 456 eV (blue). (**e**) High resolution XPS spectra for the Ti 2p core level of TiO2 and 1%, 2% Cu2O/TiO2 nanocomposites.

Cu_2_O/TiO_2_ nanocomposites with different Cu_2_O loading, between 0.5% and 5%, were synthesized by the hydrothermal reaction of copper (II) nitrate hexahydrate and titanium (IV) isopropoxide at 170 °C for 12 h. Figure 1a shows the XRD patterns of the TiO_2_ and Cu_2_O/TiO_2_ nanopowders. The main XRD peaks of all patterns could be indexed with the tetragonal anatase TiO_2_ phase (JCPDS No. 01-071-1167). Additional XRD peaks at 2θ = 36.4° and 42.3° were identified in the Cu_2_O/TiO_2_ samples containing 1% and higher Cu_2_O amounts and were associated with the (111) and (200) family planes of the cubic Cu_2_O cuprite phase. From the XRD patterns, using the Scherrer equation, the size of the TiO_2_ and Cu_2_O crystal domains was calculated to be ca. 7 nm and 50 nm, respectively, which pointed at the presence of some large Cu_2_O crystals.

TEM micrographs showed TiO_2_ and Cu_2_O/TiO_2_ nanopowders that consisted of small nanoparticles with irregular shapes and an average size of ca. 10 nm (Figure 1b and Supplementary Figure S2). HRTEM characterization of the 1% composite further confirmed the presence of both the tetragonal anatase TiO_2_ and cubic Cu_2_O phases (Figure 1c). STEM-EELS compositional map displayed the elemental distribution (Figure 1d). By performing the quantitative relative compositional analysis, we could extrapolate that the Ti and O compositions oscillate between 30–35% and 65–70%, respectively. Only traces of Cu could be detected from the 1% composite (TS1). This limitation and the small size of the Cu_2_O domains observed by HRTEM resulted in a STEM-EELS compositional map showing homogeneous-like copper distributions (Figure 1d). SEM-EDX analysis showed the Cu concentration to match the nominal amount in low Cu-loaded samples but to be lower than expected in 2% and 5% Cu_2_O/TiO_2_ nanocomposites (Table S1).

XPS spectra showed the incorporation of Cu not to influence the Ti chemical state (Figure 1e and Supplementary Figure S3), which displayed the Ti 2p_3/2_ and Ti 2p_1/2_-binding energies at 458.5 eV and 464.2 eV, respectively, consistent with Ti^4+^ within a TiO_2_ chemical environment [27,28,29]. Besides, the Cu 2p_3/2_ and Cu 2p_1/2_-binding energies were 931.9 eV and 951.9 eV, pointing at a Cu^+^ chemical state [30,31]. The surface composition of Cu matched the nominal amount of Cu in the 1% Cu_2_O/TiO_2_ nanocomposite, but it was lower for the 2% Cu_2_O/TiO_2_ nanocomposite, which is, in part, consistent with SEM-EDX analysis and, in part, associated to the formation of relatively large Cu_2_O particles when increasing the Cu loading, as observed by XRD.

**Figure 2:**
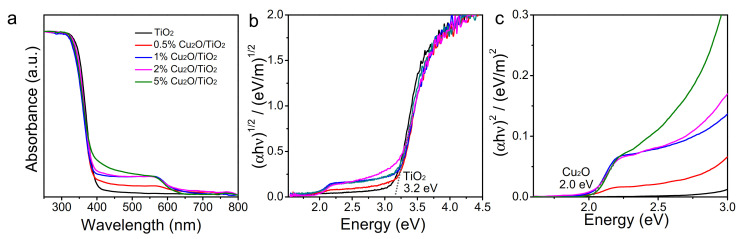
UV-vis absorption spectra (**a**) and Tauc plot calculated as (αh*ν*)^1/2^ vs. h*ν* (**b**) and as (αh*ν*)^2^ vs. h*ν* t (**c**) for TiO_2_ and 0.5%, 1%, 2% and 5% Cu_2_O/TiO_2_ nanocomposites.

Figure 2 shows the UV-vis spectra of TiO_2_ and Cu_2_O/TiO_2_ nanopowders and the corresponding Tauc plot calculated as (αh*ν*)^1/2^ vs. h*ν* to determine the direct bandgap of TiO_2_ (Figure 2b) and, as (αh*ν*)^2^ vs. h*ν*, to determine the indirect bandgap of Cu_2_O (Figure 2c). UV-vis absorption data showed a clear absorption edge at around 3.2 eV consistent with the TiO_2_ bandgap. No clear shift of the absorption edge was observed with the introduction of Cu, which ruled out a possible bandgap change related to the incorporation of Cu ions within the TiO_2_ lattice. Besides, when incorporating Cu_2_O, additional light absorption in the visible region and with an absorption edge of ca. 2.0 eV was clearly observed, consistent with the presence of the Cu_2_O phase [32].

### 3.2. Photocatalytic Activity

**Figure 3:**
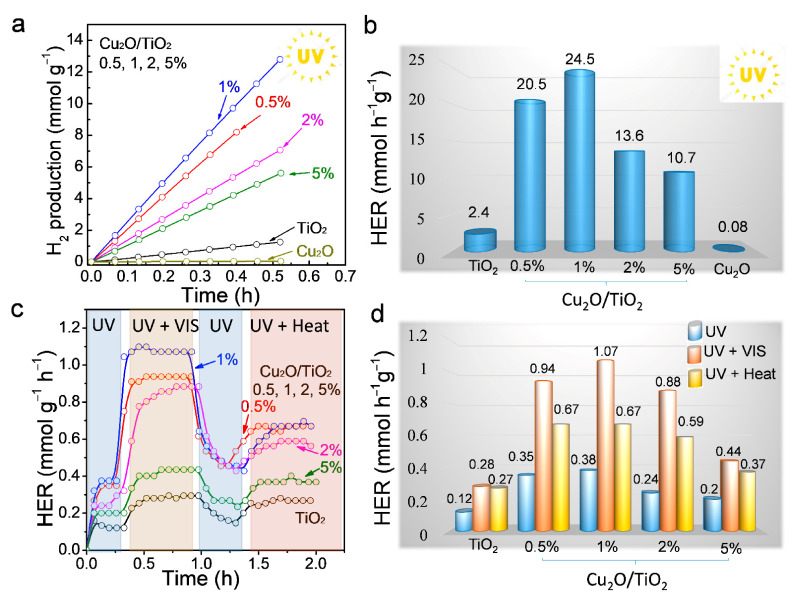
(**a**) Photocatalytic H_2_ evolution on TiO_2_, Cu_2_O, 0.5%, 1%, 2% and 5% Cu_2_O/TiO_2_ nanocomposites under UV light irradiation (365 ± 5 nm and 79.1 ± 0.5 mW·cm^−2^). (**b**) HER from data displayed in panel (**a**). (**c**) HER measured on TiO_2_, 0.5%, 1%, 2% and 5% Cu_2_O/TiO_2_ nanocomposites under different conditions: (1) UV light irradiation (372 ± 5 nm and 11.2 ± 0.5 mW·cm^−2^), (2) UV (372 ± 5 nm and 11.2 ± 0.5 mW·cm^−2^) plus visible light irradiation (0.017 ± 0.005 mW·cm^−2^), (3) UV light irradiation and (4) UV light irradiation and heating to compensate for the temperature (~36−37 °C). (**d**) HER obtained from the data displayed in panel (**c**).

Figure 3a,b displays the UV (365 ± 5 nm) photocatalytic activity of TiO_2_, Cu_2_O/TiO_2_ and Cu_2_O nanopowders toward hydrogen production from a gas phase 10% ethanol solution in water. The composition of the effluent gas was monitored using gas chromatography, which showed acetaldehyde (2) and hydrogen (3) in a 1:1 molar ratio to be the two unique products of the reaction. These results proved both that the hydrogen was generated from the dehydrogenation of ethanol and not from water splitting and that the reaction proceeded with very high selectivity toward acetaldehyde production, following the scheme [33]:

(2) $${\mathrm{CH}}_{3}{\mathrm{CH}}_{2}\mathrm{OH}+2h^{+}\;\to {\;\mathrm{CH}}_{3}\mathrm{CHO}+2H^{+}$$

(3) $$2H^{+}+2e^{-}\to {\;H}_{2}$$

The hydrogen evolution rate (HER) measured under UV light for the reference TiO_2_ was 2.4 mmol h^−1^·g^−1^ (Figure 3a,b). HER strongly increased with the introduction of Cu_2_O (Table S2). Among the series of Cu_2_O/TiO_2_ samples tested, the highest HRE were obtained for the 0.5% and 1% Cu_2_O/TiO_2_ samples that displayed a HER of 20.5 mol∙g^−1^∙h^−1^ and 24.5 mmol∙h^−1^∙g^−1^, a factor of 10 above bare TiO_2_. Higher Cu_2_O loadings that resulted in lower HER, 13.6 and 10.7 mmol∙g^−1^∙h^−1^ for the 2% and 5% samples, respectively. We hypothesize the lower HER obtained when increasing the Cu loading above 1% to be related with an increase of the recombination rate associated with a faster recombination of the charge carriers photogenerated in the Cu_2_O phase than in the TiO_2_ phase. Besides, the formation of larger Cu_2_O domains when increasing the Cu loading could also play an important role. The AQY of the 1% Cu_2_O/TiO_2_ was 6.4%, whereas the AQY for TiO_2_, 0.5%, 2% and 5% Cu_2_O/TiO_2_ were 0.6%, 5.3%, 3.5% and 2.8%, respectively (Figure S4). Table S2 displays a comparison of the AQY obtained here with those obtained in previous works. On the other hand, the HER of bare Cu_2_O was very moderate, just 0.8 mmol∙h^−1^∙g^−1^, demonstrating both the important role played by TiO_2_ in the separation of charge carriers and the synergism between the two materials to optimize photocatalytic activity. Figure S5 displays the HER of the 1% Cu_2_O/TiO_2_ sample, measured three consecutive times during 1 h, showing the notable HER stability of the system. Besides, in contrast to some previous works, we observed no color change of our samples during the photocatalytic reaction in the presence of ethanol [34,35]. It should be noted, that beyond the convenient use of aqueous ethanol solutions, as produced from biomass processing, the presence of water is beneficial to increase of the catalyst activity and stability by preventing active sites to be blocked by acetaldehyde, which exhibits a strong affinity towards inorganic oxide surfaces [36].

Figure 3c shows the photocatalytic HER activities of Cu_2_O/TiO_2_ under UV light (372 ± 5 nm) and when combining UV light with visible light or heat (see the experimental section for details). It should be noted that, under visible light, there is an increase in the temperature of the photocatalyst; thus, it is necessary to separate the effect on HER of the temperature increase and the photogenerated charge carriers obtained with the visible light absorption. Thus, the photocatalytic test was divided into four consecutive steps: (i) After turning on the UV light, HER began to rise until it stabilized. At this stage, the sample temperature was ca. 25 °C. (ii) Keeping the UV light on, the visible light was turned on, which increased the HER of all samples. The introduction of visible light also increased the sample temperature, up to ca. 36–37 °C (Table S3). (iii) With the UV light on, the visible light was turned off, which resulted in a relatively slow decline of the HER and a temperature decrease down to 25 °C. The slow HER decrease already denoted a significant effect of temperature on the increase of HER observed with the visible light. (iv) Finally, still maintaining the UV light on, the reactor was heated to 36–37 °C (Table S3), which also resulted in an increase of the HER for all catalysts. By comparing stages 2 and 4, the effect of temperature and photogenerated electron–hole pairs can be differentiated.

Notice that the addition of visible light increased the HER of TiO_2_ by a factor of two, which was associated with a 10 °C increase in temperature (Figure 3c,d). This twofold HER increase points toward the combination of energy sources in thermo-photocatalytic reactors as an efficient strategy of solar energy conversion. Such a strong influence of temperature on HER is likely related to the high adsorption energy of acetaldehyde on the oxide surface, blocking the catalyst active sites and, thus, slowing down the reaction. A moderate increase in temperature can significantly reduce the acetaldehyde adsorption strength, thus unblocking active sites and increasing the activity [37].

Cu_2_O/TiO_2_ catalysts displayed a much higher increase of activity with the addition of visible light (Figure S6) by close to a factor of three in 1% Cu_2_O/TiO_2_. Only a small fraction of this increase in activity can be associated with the increase of temperature, as observed in Figure 3c,d. The much larger increase of HER obtained with visible light irradiation compared to the sample heating to the same temperature suggests a significant contribution of photogenerated charge carriers in Cu_2_O.

### 3.3. Photoluminescence and Photoconductivity

**Figure 4:**
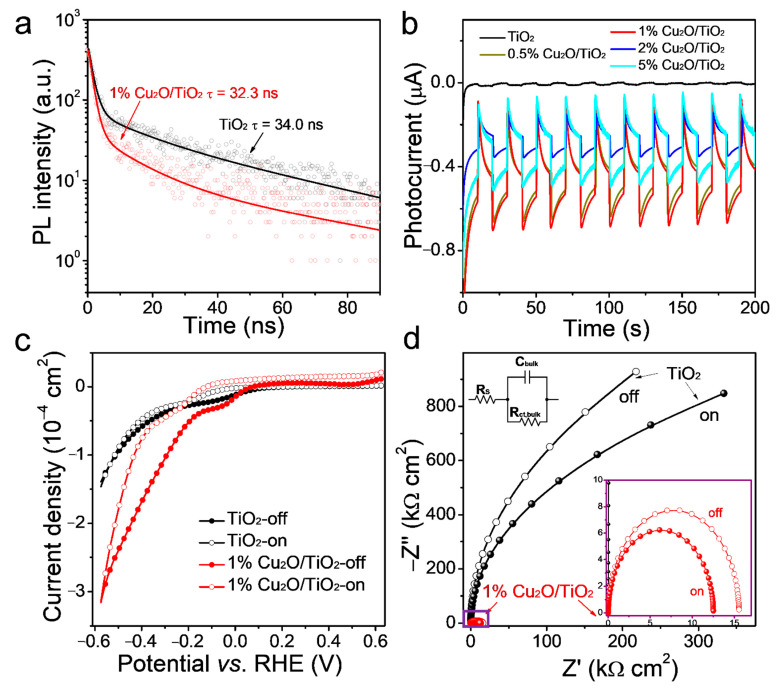
(**a**) TRPL decay of the TiO_2_ and 1% Cu_2_O/TiO_2_ composites. (**b**) Transient photocurrent response for TiO_2_ and 0.5%, 1%, 2% and 5% Cu_2_O/TiO_2_ composites. (**c**) Current density vs. potential (RHE) and (**d**) Nyquist plot with the EIS data obtained from TiO_2_ and the 1% Cu_2_O/TiO_2_ composite in the dark (off) and under illumination (on) at the AM1.5G solar power system 100 mW·cm^−2^ light irradiation.

The photocatalytic performance of the semiconductor photocatalyst is tightly related to their charge transport, separation and transfer processes, which closely rely on their relative electronic energy level positions. To understand the photocatalytic process and to gain insights from the enhanced performances of the Cu_2_O/TiO_2_ nanocomposites, a series of spectroscopic analyses was performed. Figure S7 displays the steady-state PL spectra of TiO_2_ and 1% Cu_2_O/TiO_2_. In both spectra, a peak at around 400 nm, associated with the band-to-band radiative recombination in TiO_2_, was observed [38,39,40,41,42]. The presence of Cu_2_O resulted in a decrease of the peak intensity, which denoted an influence of Cu_2_O on the recombination of charge carriers photogenerated in TiO_2_. Figure 4a displays the TRPL spectra of TiO_2_ and 1% Cu_2_O/TiO_2_ at 400 nm. The PL intensity of both samples was observed to decay at a similar rate, with an average photocarrier lifetime of 32.3 ns for 1% Cu_2_O/TiO_2_ and 34.0 ns for TiO_2_. This result demonstrated a minor influence of Cu_2_O on the band-to-band recombination within TiO_2_, thus pointing again toward a minor or null influence of Cu within the TiO_2_ lattice.

The photoelectrochemical behavior of TiO_2_ and 1% Cu_2_O/TiO_2_ samples supported on an ITO-covered glass substrate were measured under dark and 100-mW·cm^−2^ AM 1.5G irradiation. As shown in Figure 4c, the photocurrent density measured for 1% Cu_2_O/TiO_2_ was higher than that obtained for TiO_2_. Figure 4b displays the TPC data obtained from the TiO_2_ and Cu_2_O/TiO_2_ composites with different Cu_2_O loadings. The 1% Cu_2_O/TiO_2_ electrode showed the highest photocurrent densities, well above those obtained for bare TiO_2_. The stable photocurrent of all Cu_2_O/TiO_2_ samples pointed at a good stability of the composites under illumination in the solution. The 5% Cu_2_O/TiO_2_ sample showed the largest TPC transient spikes, indicating the highest degree of surface charge recombination, which is consistent with its lower HER catalytic performance (Figure 3b) and suggests that an excessive amount of Cu_2_O hampers the photocatalytic activity due to excessive charge carrier recombination [43].

Figure 4d displays the Nyquist plot with the EIS data obtained from TiO_2_ and 1% Cu_2_O/TiO_2_ in the dark and under illumination. EIS analysis showed the 1% Cu_2_O/TiO_2_ sample to be much less resistive than TiO_2_ [44], suggesting that the formation of the heterojunction facilitates the charge transport and injection. Data were fitted with a Randles equivalent circuit consisting of a series resistor $R_{S}$, a bulk resistor $R_{ct,bulk}$ for charge transport resistance and a bulk capacitor $C_{bulk}$ for space charge region capacitance (Table S4) [45]. With the incorporation of only 1% of Cu_2_O, the value of $R_{ct,bulk}$ was reduced from 4.18 Ω to 15.5 Ω in the dark and to even lower values under AM1.5G irradiation.

### 3.4. Determination of Heterojunction Band Position

To determine the band alignment of the Cu_2_O/TiO_2_ heterojunction; the M–S analysis was performed on pristine TiO_2_, Cu_2_O and 1% Cu_2_O/TiO_2_, considering:

(4) $$C^{-2}=\frac{2}{N_{D}{\varepsilon \varepsilon }_{0}e}\left(V-V_{f_{b}}-\frac{\mathrm{kT}}{e}\right)$$

where C is the space charge capacitance in the semiconductor, $N_{D}$ is the electron carrier density, e is the elementary charge (1.60 × 10^−19^ C) and $\varepsilon_{0}$ is the vacuum permittivity (8.85 × 10^−12^ F∙m^−1^). The considered relative permittivity was ε = 55 for TiO_2_ and ε = 6.3 for Cu_2_O [46].

**Figure 5:**
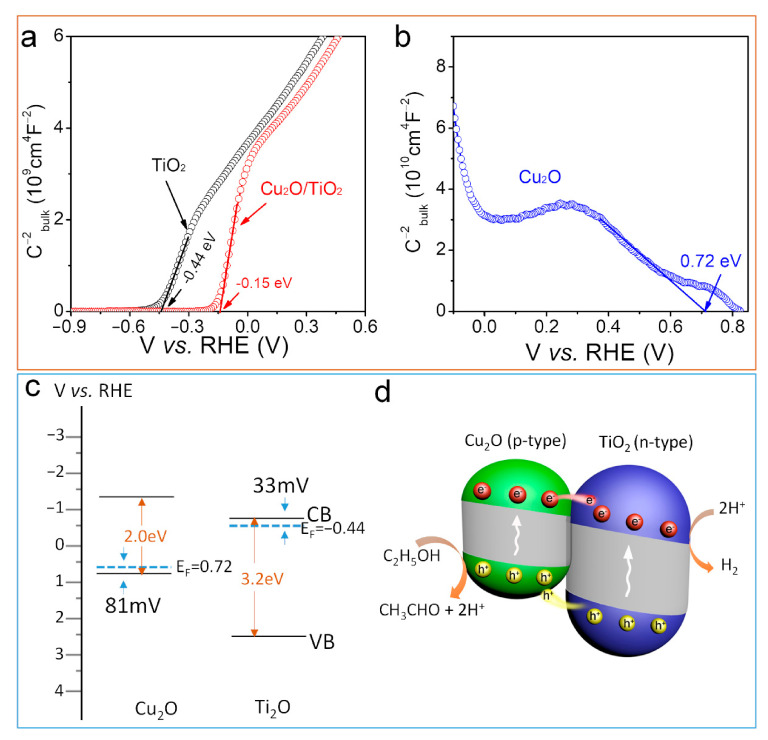
(**a**) M-S analysis of TiO_2_ and 1% Cu_2_O/TiO_2_. (**b**) M-S analysis of a Cu_2_O. (**c**) Energy band diagrams for Cu_2_O and TiO_2_ before contact. (**d**) Scheme of the Energy band structure of a Cu_2_O/TiO_2_ heterojunction and the ethanol dehydrogenation reaction.

Figure 5a shows the M–S plots of TiO_2_, Cu_2_O and 1% Cu_2_O/TiO_2_. $N_{D}$ is determined as:

(5) $$N_{D}=\frac{2}{{e\varepsilon }_{0}\varepsilon }\times {\left[\frac{d\left[\frac{1}{C^{2}}\right]}{{\mathrm{dv}}_{s}}\right]}^{-1}$$

where ${\left[\frac{d\left[\frac{1}{C^{2}}\right]}{dv_{s}}\right]}^{-1}$ is the best fit of their linear range of $\left[\frac{1}{C^{2}}\right]$ vs. V (12 × 10^9^ cm^4^ ∙F^−2^ for TiO_2_ and 9 × 10^10^ cm^4^∙ F^−2^ for Cu_2_O). As expected, TiO_2_ shows a positive value in the linear region in accordance with its n-type character, while Cu_2_O shows a negative value consistent with its p-type behavior [30]. The M–S analysis resulted in $N_{D}$ = $2.14\times 10^{20}{\;\mathrm{cm}}^{-3}$ for TiO_2_ and $N_{D}$ = $2.5\times 10^{20}{\mathrm{cm}}^{-3}$ for Cu_2_O.

The effective density of states in the conduction band ($N_{C}$) is given by:

(6) $$N_{C}\equiv 2{\left(\frac{2{\pi m}_{\mathrm{de}}\mathrm{kT}}{h^{2}}\right)}^{\frac{3}{2}}$$

where m_de_ is the density-of-state effective mass for electrons of nano-crystalline anatase TiO_2_, h is Planck’s constant (6.62607004 × 10^−34^ m^2^∙kg∙s^−1^), k is Boltzmann’s constant (1.38064852 × 10^−23^ m^2^∙kg∙s^−2^∙K^−1^) and T is the absolute temperature (298 K). For TiO_2_ a $m_{\mathrm{de}}$=10${\;m}_{0}$ was used for Nc calculations, where${\;m}_{0}$ (9.109 × 10^−31^) is the mass of a free electron. For Cu_2_O $m_{\mathrm{de}}$= 0.58 m_0_ is taken as the effective hole mass.

Boltzmann statistics was applied to determine the position of the bottom of the conduction band $E_{\mathrm{CB}}$ for TiO_2_ and the maximum of the valence band $E_{\mathrm{VB}}$ for Cu_2_O (3):

(7) $$E-E_{F}=\mathrm{kT}\ln\left(\frac{N_{C}}{N_{D}}\right)$$

where $E_{F}$ is the Fermi level position ($E_{F}$ = $V_{\mathrm{fb}}$). $E_{F}$ was found to be 0.033 eV below the $E_{\mathrm{CB}}$ for TiO_2_ and 0.081 eV above the $E_{\mathrm{VB}}$ for Cu_2_O [47]. Based on the M–S analysis, the electronic band structure of Cu_2_O and TiO_2_ is displayed in Figure 5c. The $V_{\mathrm{fb}}$ values of TiO_2_ and Cu_2_O are −0.44 eV and 0.72 eV vs. RHE, respectively. The${\;E}_{\mathrm{CB}}$ for TiO_2_ was −0.41 eV and the $E_{\mathrm{VB}}$ for Cu_2_O is 0.64 eV. As the two materials are brought into contact, there is a net transfer of electrons from n-type TiO_2_ to p-type Cu_2_O that results in a bending of the band structure at the interface. Due to the small size of the crystal domains, this bending extends through all the whole TiO_2_ and Cu_2_O crystals that are in contact with each other. (Figure 5d). In the resulting heterostructure, photogenerated electrons in the conduction band of Cu_2_O tend to move toward TiO_2_, where hydrogen generation takes place, and photogenerated holes in TiO_2_ tend to move toward the Cu_2_O, where ethanol is oxidized to acetaldehyde (Figure 5d) [48,49].

## 4. Conclusions

A simple one-pot method for the synthesis of p-n Cu_2_O/TiO_2_ heterostructures was presented. Using UV-vis spectroscopy, and M–S analyses, we showed the formation of a p-n heterojunction between Cu_2_O and TiO_2_, which favors the separation of electron–hole pairs. They obtained nanocomposites at 0.5%, 1%, 2% and 5% Cu_2_O loading were tested for the photocatalytic dehydrogenation of ethanol in water:ethanol vapor mixture. We demonstrated the composites to be photostable catalysts capable of working in a light absorption towards the visible range, with an outstanding selectivity to the production of acetaldehyde and hydrogen from ethanol. The optimum composition contained 1% of Cu_2_O and showed a yield for HER of 24.5 mmol∙g^−1^∙h^−1^ and an AQY = 6.4%. The EIS analysis showed the 1% Cu_2_O/TiO_2_ sample to be less resistive than TiO_2_ sample and suggested that the heterojunction facilitated the charge transport and injection. The addition of visible light increased the HER of the samples by a factor of two, which was partially associated with an increment in the reaction temperature of around 10 °C. We further discerned the influence of temperature and photogenerated electron–hole pairs in the HER increase upon visible light irradiation, demonstrating the important role of photogenerated charge carriers in the presence of Cu_2_O. Besides, our results open new opportunities for efficient solar energy conversion by the combination of energy sources in thermo-photocatalytic reactors.

## Data Availability

The data is available on reasonable request from the corresponding authors.

## Supplementary Materials

The following are available online at https://www.mdpi.com/article/10.3390/nano11061399/s1, Figure S1. (a) Photograph of the system used to test photocatalytic hydrogen generation: 1 displays the flask containing the ethanol-water solution (1:9) and 2 displays the actual photoreactor. (b) Scheme of the photoreactor under UV-light, (c) Scheme of the central part of the photoreactor. Figure S2. TEM micrograph of TiO2 nanocrystals. Figure S3. (a) Survey XPS spectra of TiO2 and 1% and 2% Cu2O/TiO2 nanocomposites. (b) High-resolution XPS spectra for Cu 2p core level of 1% and 2% Cu2O/TiO2 nanocomposites. Figure S4. AQY data of HER obtained on TiO2 and 0.5%, 1%, 2%, 5% Cu2O/TiO2 nanocomposites. Figure S5. Three consecutive cycles of photocatalytic hydrogen production under UV light using the same 1% Cu2O/TiO2 sample. Figure S6 Emission spectrum of the visible LED used for visible illumination recorded by an ocean optics spectrometer (USB2000+XR1-ES). Figure S7. Photoluminescence spectra of TiO2 (black line) and 1% Cu2O/TiO2 nanocomposite (red line). Table S1. Ti and Cu atomic concentrations of 0%, 0.5%, 1%, 2%, 5% Cu2O/TiO2 nanocomposites. Table S2. Comparison of the hydrogen evolution rates reported on Cu2O-TiO2 systems. Table S3. Temperature evolution in the photocatalytic reaction of the 0%, 0.5%, 1%, 2%, 5% Cu2O/TiO2 nanocomposites. Table S4. EIS data fitting results obtained from TiO2 and 1% Cu2O/TiO2 nanocomposite in the dark (off) and under 100 mW·cm-2 AM 1.5G irradiation (on).
