# Supplementary material for: Photodehydrogenation of Ethanol over Cu2O/TiO2 Heterostructures
Source: Nanomaterials (Basel). 2021 May 25;11(6):1399. doi: 10.3390/nano11061399 (PMC8230259; doi:10.3390/nano11061399)
Supplement: Supplementary file 1 [file nanomaterials-11-01399-s001.zip › nanomaterials-1221268-supplementary.pdf]

Supplementary Materials

Photodehydrogenation of Ethanol over Cu_2O/TiO_2 Heterostructures

Congcong Xing,[1,2] Yu Zhang,[1] Yongpeng Liu,[3] Xiang Wang,[1] Junshan Li,[1] Paulina R. Martínez-Alanis,[4] Maria Chiara Spadaro,[5] Pablo Guardia,[1] Jordi Arbiol,[5,6] Jordi Llorca,*[2] and Andreu Cabot.*[1,6]

[1] Catalonia Institute for Energy Research (IREC), Sant Adrià de Besòs, 08930 Barcelona, Spain; congcongxing@irec.cat (C. X.), peterzhang@irec.cat (Y. Z.), wxiang@irec.cat (X. W.), junshanli@irec.cat (J. L.), pguardia@irec.cat (P. G.)

[2] Institute of Energy Technologies, Department of Chemical Engineering and Barcelona Research Center in Multiscale Science and Engineering, Universitat Politècnica de Catalunya, EEBE, 08019 Barcelona, Spain

[3] Laboratory for Molecular Engineering of Optoelectronic Nanomaterials (LIMNO), École Polytechnique Fédérale de Lausanne (EPFL), Station 6, CH-1015 Lausanne, Switzerland; yongpeng.liu@epfl.ch (Y. L.)

[4] ENFOCAT-IN2UB, Universitat de Barcelona (UB), C/Martí i Franquès 1, 08028 Barcelona, Catalunya, Spain; paulina.martinez@ub.edu (P.R.M.A.)

[5] Catalan Institute of Nanoscience and Nanotechnology (ICN2), CSIC and BIST, Campus UAB, Bellaterra, 08193 Barcelona, Catalonia, Spain; mariachiara.spadaro@icn2.cat (M. C. S.)

[6] ICREA, Pg. Lluis Companys 23, 08010 Barcelona, Spain; jordi.arbiol@icn2.cat (J. A.)

* E-mails: A. Cabot: acabot@irec.cat; J. Llorca: jordi.llorca@upc.edu.

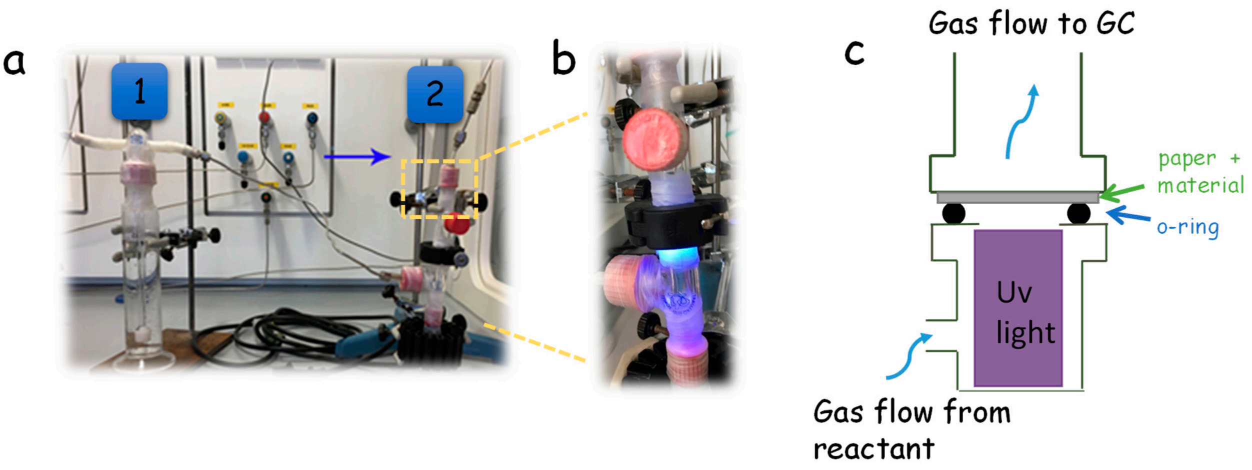

Figure S1. (a) Photograph of the system used to test the photocatalytic hydrogen generation: *1* displays the flask containing the ethanol-water solution (1:9) and *2* displays the actual photoreactor. (b) Scheme of the photoreactor under UV-light, (c) Scheme of the central part of the photoreactor.

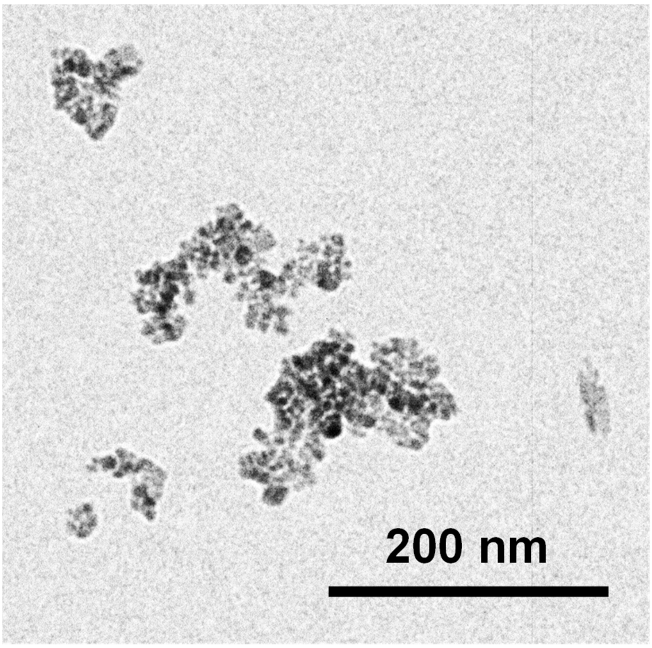

FigureS2. Representative TEM micrograph of TiO_2 nanocrystals.

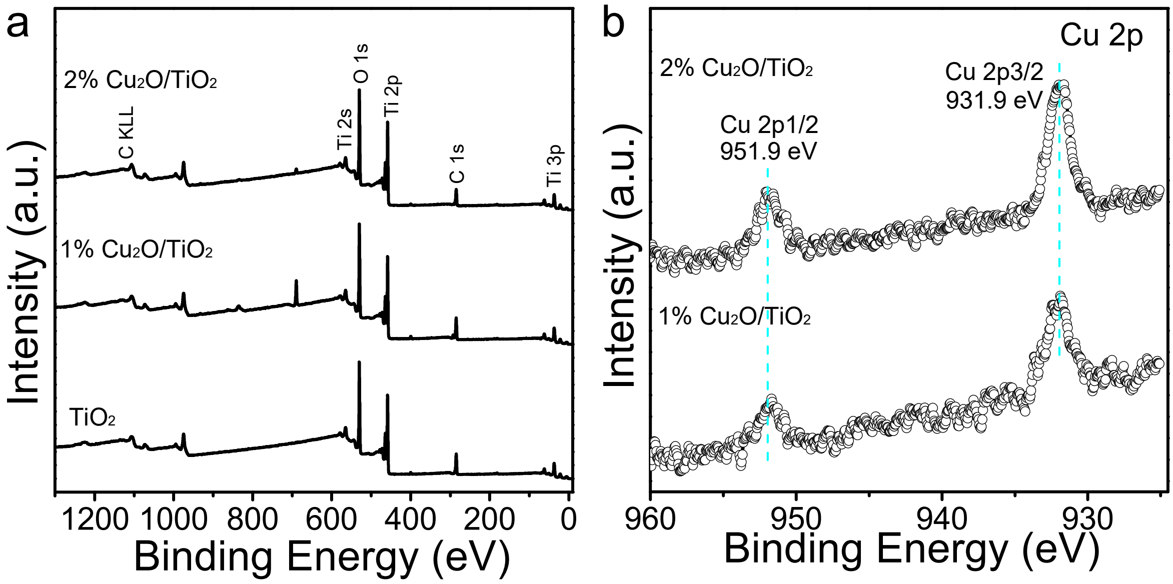

FigureS3. (a) Survey XPS spectra of TiO_2 and 1% and 2% Cu_2O/TiO_2 nanocomposites. (b) High-resolution XPS spectra for Cu 2p core level of 1% and 2% Cu_2O/TiO_2 nanocomposites.

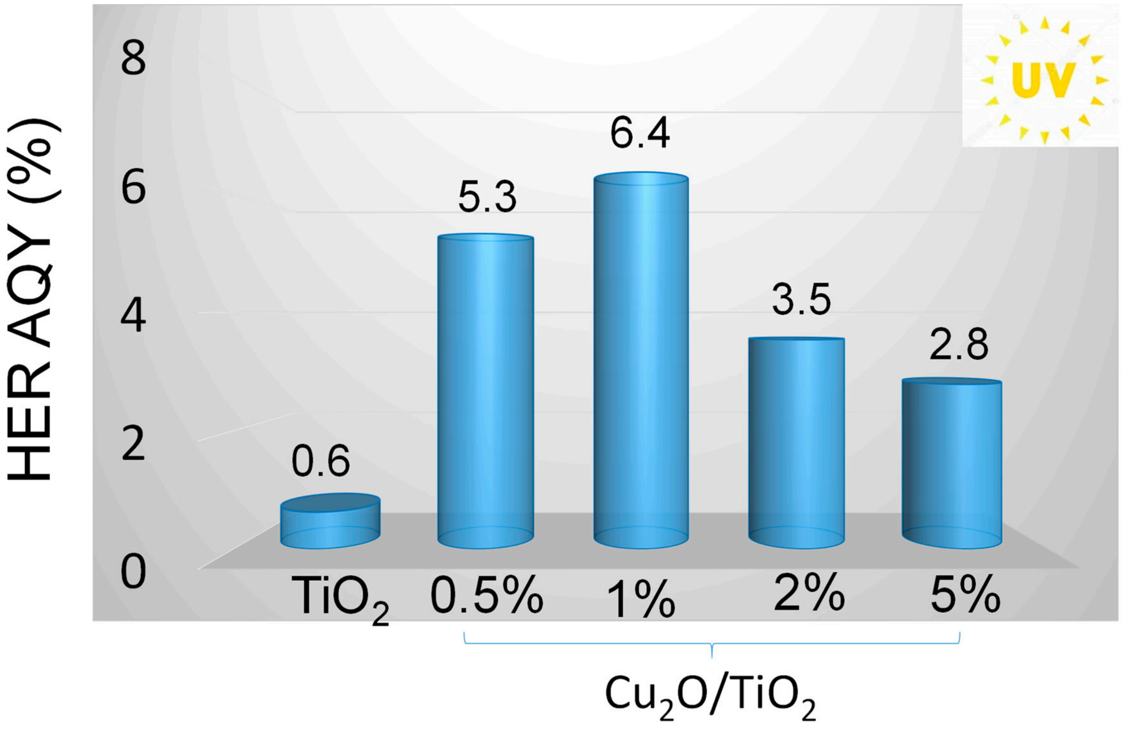

Figure S4. Aparent quantum yield (AQY) of the hydrogen evolution rate (HER) obtained on TiO_2 and 0.5%, 1%, 2%, 5% Cu_2O/TiO_2 nanocomposites.

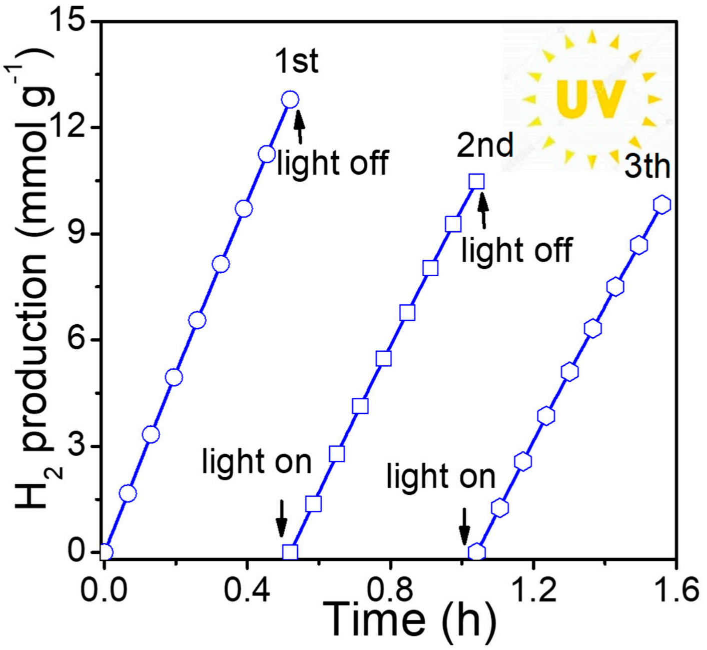

Figure S5. Three consecutive cycles of photocatalytic hydrogen production under UV light using the 1% Cu_2O/TiO_2 sample.

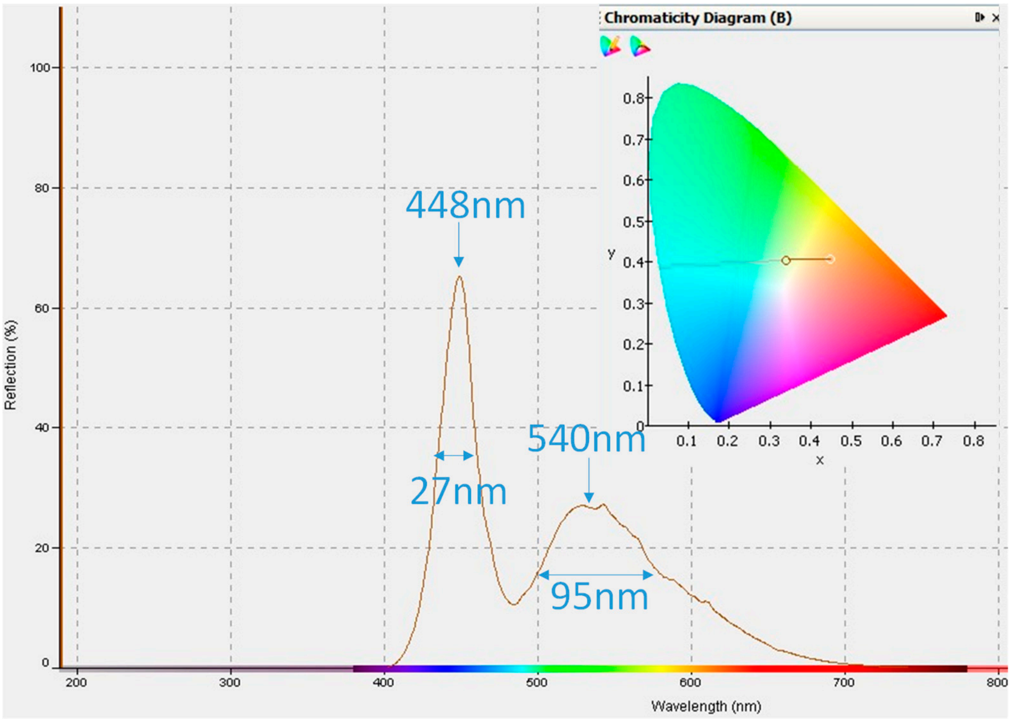

Figure S6. Emission spectrum of the visible LED used for visible illumination recorded using an ocean optics spectrometer (USB2000+XR1-ES). Inset shows the LED chromaticity diagram.

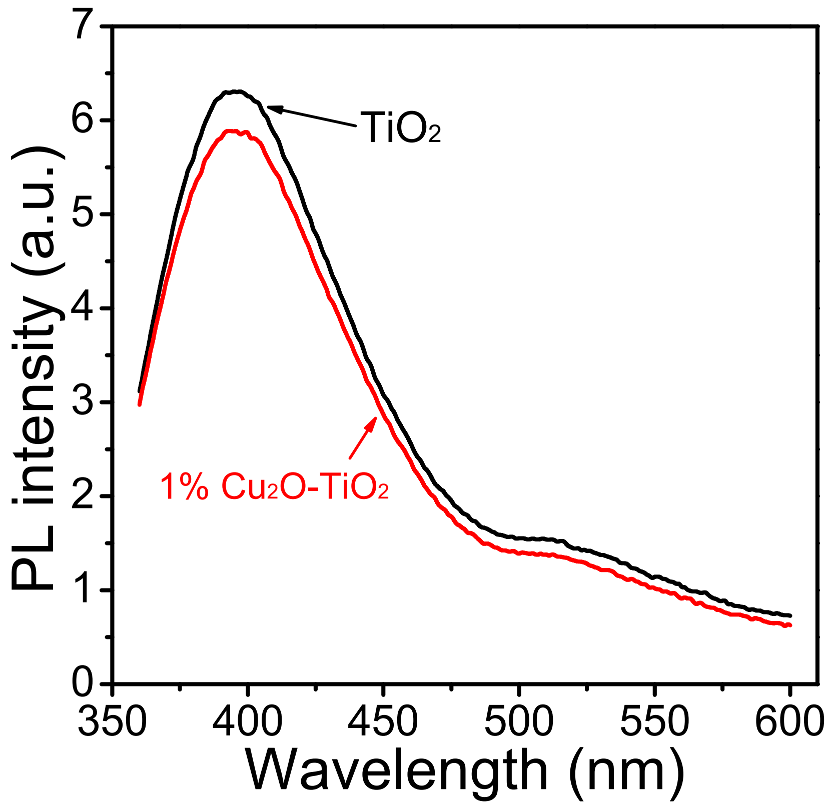

Figure S7. Steady-state photoluminescence (PL) spectra of TiO_2 and 1% Cu_2O/TiO_2 nanocomposite. The PL spectra were recorded on a high resolution PL spectrofluorometer (Horiba Jobin Yvon Fluorolog-3) with an exitation wavelength of 350 nm.

Table S1. Ti and Cu atomic concentrations of 0%, 0.5%, 1%, 2%, 5% Cu_2O/TiO_2 nanocomposites.

Catalyst	EDX		XPS	
	Ti (at%)	Cu (at%)	Ti (at%)	Cu (at%)
TiO_2	100		100	
0.5% Cu_2O/TiO_2	99.4	0.6		
1% Cu_2O/TiO_2	99.1	0.9	98.9	1.1
2% Cu_2O/TiO_2	98.8	1.2	99.0	1.0
5% Cu_2O/TiO_2	97.1	2.9		

Table S2. Comparison of the hydrogen evolution rates reported on Cu_2O-TiO_2 systems.

Photocatalyst	H_2 Evolution Rate μmol h^{-1} g^{-1}	Illumination Light	AQY	Ref.
5*wt*%Cu/Cu_2O/Cu/TiO_2	3,944	520 nm; 100 mW cm^{-2}	45.7%	S1
0.9 mol% Cu_2O/TiO_2	318	365 nm	28.6%	S2
Cu_2O ca.1.5% (Cu_2O /T1-V_o)	326,000	350 nm	53.5%	S3
Ag/Cu@Cu_2O	874.7	365 nm	2.3%	S4
2.5 mol% Cu_2O/TiO_2	2,048	365 nm; 3.6 mWcm^{-2}	4.32%	S5
Cu/Cu_2O/CuO/TiO_2	2,008	100 mW cm^{-2}	2.21%	S6
1% Cu_2O/TiO_2	24,500	365nm; 79.1 mW cm^{-2}	6.4%	This work

Table S3. Temperature evolution in the photocatalytic reaction of the 0%, 0.5%, 1%, 2%, 5% Cu_2O/TiO_2 nanocomposites.

Sample	UV Temperature (°C)	UV+Vis Temperature (°C)	UV+heating Temperature (°C)
TiO_2	24	35	35
0.5% Cu_2O/TiO_2	24	37	37
1% Cu_2O/TiO_2	25	36	36
2% Cu_2O/TiO_2	24	35	35
5% Cu_2O/TiO_2	25	37	37

Table S4. EIS data fitting results obtained from TiO_2 and 1% Cu_2O/TiO_2 nanocomposite in the dark (off) and under 100 mW·cm^{-2} AM 1.5G irradiation (on).

Photocatalyst	$R_{ct,bulk}$ (kΩ)	C_{bulk} (μF)	Rs (Ω)
TiO_2-off	4179	13.41	29.34
TiO_2-on	2492	13.42	29.44
1%Cu_2O/TiO_2-off	15.52	13.73	26.41
1%Cu_2O/TiO_2-on	12.48	13.04	27.46

Refrerences

S1. Zhen, W.; Jiao, W.; Yuqi Wu.; Jing, H.; Lu, G. The Role of a Metallic Copper Interlayer during Visible Photocatalytic Hydrogen Generation over a Cu/Cu_2O/Cu/TiO_2 Catalyst. *Cite this Catal. Sci. Technol* **2017**, *7*, 5028. doi:10.1039/c7cy01432e.

S2. Li, L.; Xu, L.; Shi, W.; Guan, J. Facile Preparation and Size-Dependent Photocatalytic Activity of Cu_2O Nanocrystals Modified Titania for Hydrogen Evolution. *Int. J. Hydrog. Energy* **2012**, 38, 816-822. doi:10.1016/j.ijhydene.2012.10.064.

S3. Wei, T.; Zhu, Y.-N.; An, X.; Liu, L.-M.; Cao, X.; Liu, H.; Qu, J. Defect Modulation of Z-Scheme TiO_2/Cu_2O Photocatalysts for Durable Water Splitting. *ACS Catal.* **2019**, *9*, 8346–8354**.**doi:10.1021/acscatal.9b01786.

S4. Fu, J.; Cao, S.; Yu, J. Dual Z-Scheme Charge Transfer in TiO_2–Ag–Cu_2O Composite for Enhanced Photocatalytic Hydrogen Generation. *J. Mater.* **2015**, *1* (2), 124–133. doi:10.1016/j.jmat.2015.02.002.

S5. Li, Y.; Wang, B.; Liu, S.; Duan, X.; Hu, Z. Synthesis and Characterization of Cu_2O/TiO_2 Photocatalysts for H_2 Evolution from Aqueous Solution with Different Scavengers. *Appl. Surf. Sci.* **2015**, *324*, 736–744. doi:10.1016/j.apsusc.2014.11.027

S6. Zhang, Z.; Liu, K.; Bao, Y.; Dong B. Photo-assisted self-optimizing of charge-carriers transport channel in the recrystallized multi heterojunction nanofibers for highly efficient photocatalytic H_2 generation. *Appl. Catal. B Environ.* **2017**, *203*, 599–606. doi:10.1016/j.apcatb.2016.10.064
